# Corifollitropin alfa compared to daily FSH in controlled ovarian stimulation for in vitro fertilization: a meta-analysis

**DOI:** 10.1186/s13048-015-0160-4

**Published:** 2015-06-03

**Authors:** Stefania Fensore, Marco Di Marzio, Gian Mario Tiboni

**Affiliations:** Unità di Statistica, Dipartimento di Scienze Filosofiche, Pedagogiche ed Economico-Quantitative, University “G. d’Annunzio” of Chieti-Pescara, Pescara, Italy; Dipartimento di Medicina e Scienze dell’Invecchiamento, University “G. d’Annunzio” of Chieti-Pescara, Chieti, Italy

**Keywords:** Corifollitropin alfa, Recombinant FSH, Controlled ovarian stimulation, In vitro fertilization, Assisted reproductive technologies, Meta-analysis

## Abstract

The present study offers a meta-analysis of published randomized controlled trials (RCTs) evaluating the outcomes of in vitro fertilization (IVF) cycles using corifollitropin alfa for controlled ovarian stimulation (COS) in comparison with daily recombinant FSH (rFSH). The study examined seven RCTs including 2138 patients receiving corifollitropin alfa and 1788 women receiving daily rFSH for COS. As a novel aspect, this meta-analysis included two specific subpopulations of IVF patients, i.e. egg donors and poor responders. There were no significant differences between corifollitropin alfa and rFSH with respect to the majority of the clinical parameters considered, and comparable were the outcomes in terms of live birth rate, ongoing pregnancy rate, and clinical pregnancy rate. Women receiving corifollitropin alfa had a significantly higher number of metaphase II oocytes at ovum pick-up, and number of formed embryos, in comparison to rFSH. The risk of cycle cancellation due to overstimulation was significantly higher in the corifollitropin alfa group. Ovarian hyperstimulation syndrome (OHSS) incidence was statistically comparable between patients receiving long lasting or daily rFSH. Nevertheless, in view of the fact that corifollitropin alfa resulted in a higher number of metaphase II oocytes collected and a higher number of cycles cancelled due to overstimulation, corifollitropin alfa should be cautiously considered in women with the potential of being hyper responders.

## Introduction

In recent years, attention has been increasingly paid - in the field of IVF - to the development of simplified treatment approaches with the aim of reducing treatment burden and to prevent drop-out rate after a failed IVF cycle [1–3].

Corifollitropin alfa is a novel recombinant fertility hormone with prolonged follicle-stimulating activity used for COS in IVF [4]. A single subcutaneous injection of corifollitropin alfa has the capacity to initiate and sustain multiple follicular growth for the first seven days of COS, thus reducing the number of injections required during one treatment cycle [4, 5]. The long-acting properties of this new drug have been obtained by incorporating the carboxy-terminal peptide from the beta subunit of human chorionic gonadotropin (hCG) to the beta subunit of FSH [6, 7]. As unique pharmacokinetic properties, corifollitropin alfa displays a slower absorption and a longer elimination half-time in comparison to rFSH. Corifollitropin alfa has indeed a time interval to peak serum levels that is almost 4-fold longer, and the elimination half-time (approximately 69 h) that is about two-fold longer than the conventional rFSH [4, 5, 8]. Corifollitropin alfa has identical pharmacodynamics properties to rFSH since it interacts exclusively with the FSH receptor and is devoid of LH activity [5]. Based on pharmacokinetics studies and modeling [7, 9], the optimal corifollitropin doses have been identified to be: 100 μg in women who weigh less than or equal to 60 kg and who are 36 years of age or younger; 150 μg in women who weigh more than 60 kg regardless of age and women who weigh 50 kg or more and who are older than 36 years of age. The effectiveness and safety of corifollitropin alfa in comparison to rFSH have been the subject of several randomized controlled trials (RCTs). Previous meta-analyses [10, 11] have performed analysis on studies published up to 2011. The present paper aims to provide an updated meta-analysis on the corifollitropin alfa in IVF.

## Meterials and methods

### Search strategy

A systematic search of the electronic literature through MEDLINE and EMBASE databases was performed to identify studies published between 2001 and December 2014. The following headings and text strings were used alone or in combination: corifollitropin alfa, long acting FSH, in vitro fertilization, assisted reproductive technologies, *in vitro* fertilization-embryo transfer, intracytoplasmic sperm injection (ICSI). In addition, the Google Scholar database was similarly searched for studies related to corifollitropin alfa. A reference list of all included manuscripts and reviewers related to corifollitropin alfa was manually searched for additionally potentially eligible studies. Relevant journals and symposia proceedings were also evaluated to identify additional data. There was no language restriction.

### Selection criteria and data extraction

Two reviewers performed data extraction and evaluation of trial quality independently. Any disagreement concerning the extracted data was resolved by consensus and, if necessary, by involving a third reviewer. Only randomized controlled trials that compared the clinical effectiveness and safety of corifollitropin alfa with rFSH were deemed eligible for inclusion in the meta-analysis. Publications were scrutinized to identify study characteristics, randomization, allocation, blinding, and intention-to-treat analysis. Excluded studies included retrospective and uncontrolled studies, editorials and reviews. The search results were cross-checked against papers considered in previous meta-analyses [10, 11]. The target population was composed of infertile couples with any infertility factor undergoing to IVF/ICSI or egg donors, with the therapeutic intervention being corifollitropin alfa versus rFSH. The considered outcome measures were ongoing pregnancy rate, live birth and clinical pregnancy rate, early miscarriage rate, number of metaphase II oocytes per oocyte pick-up, number of embryos obtained and fertilization rate per woman with intracytoplasmic sperm injection, incidence of OHSS, adverse events, cycle cancellation and total duration. All outcomes were defined before undertaking the literature search. If additional information was required, the corresponding authors were contacted.

### Statistical analysis

Concerning the measures of the treatment effect, the present study considered both dichotomous and continuous data. As for the first, the results in the control and intervention groups of each study were expressed as Peto odds ratios (OR) with 95 % confidence intervals (CI). Continuous outcomes were expressed as weighted mean differences (WMD) with 95 % confidence intervals as well. The data were treated using fixed or random effects model. Heterogeneity of treatment effects was statistically evaluated by Hotelling T-square (τ^2^), Higgins (I^2^), Birge’s ratio (H^2^), and Chi-square test (Chi^2^). A *P-value* < 0.05 referred to the overall effect was considered statistically significant. Statistical analysis was carried out using the package *Metafor*, version 1.9–3, a free and open-source add-on for conducting meta-analyses with the statistical software environment R.

## Results

**Fig. 1:**
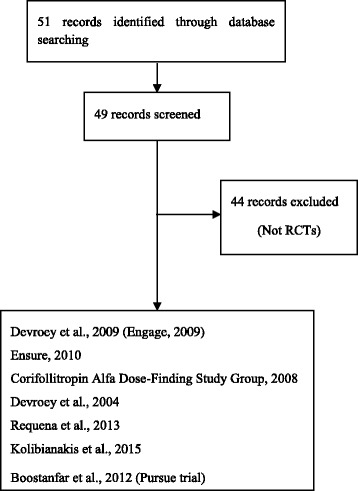
Study flow diagram

**Fig. 2:**
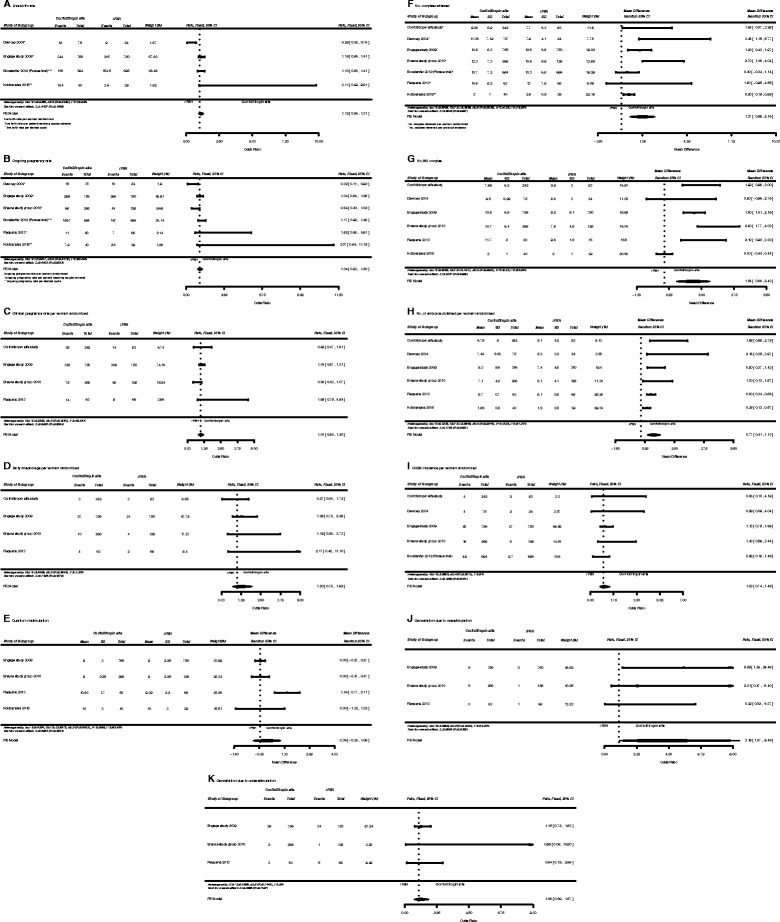
Forest plot of comparison: Corifollitropin alfa versus daily recombinant FSH. **a** Live birth rate. **b** Ongoing pregnancy rate. **c** Clinical pregnancy rate per woman randomized. **d** Early miscarriage per woman randomized. **e** Duration of stimulation. **f** No. oocytes retrieved. **g** No. MII oocytes. **h** No. of embryos obtained per woman randomized. **i** OHSS incidence per woman randomized. **j** Cancellation due to overstimulation. **k** Cancellation due to understimulation

The search yielded 51 studies of which 44 were excluded by screening through titles and abstracts (Fig. 1). Full manuscripts were retrieved for the remaining papers that included 6 full texts published in peer reviewed journals, and 1 abstract from the annual meeting of the American Society of Reproductive Medicine. Study characteristics are shown in Table 1. The seven trials enrolled a total of 3926 patients. The sample size ranged from 79 to 1506 women. In total, 2138 women were randomized to receive corifollitropin alfa and 1788 were randomized to receive daily rFSH. Results offered by the meta-analysis are shown in Fig. 2, A-K. There were differences in patient characteristics and selection. In the Engage study [12, 13] women aged 18–36 years, had a body mass index (BMI) of 18–32 kg/m^2^ and weighed 61–90 kg. In the Ensure trial [14], participants aged 18–36 years with a body weight ≤ 60 kg. In both Engage and Ensure trials patients with a history of ovarian hyper response to ovarian stimulation (more than 30 follicles ≥ 11 m) or OHSS, polycystic ovarian syndrome (PCOS) or more than 20 basal antral follicles on ultrasound (<11 mm, both ovaries combined) were excluded from the study. Women with a history of poor ovarian response were also excluded in both studies. In the Corifollitropin Alfa Dose-finding Study Group [9], patients were women aged 20–39 years with a BMI of 17–31 kg/m^2^ . In the Devroey et al. study [15], participants were between 18 and 39 year of age and a BMI 18–29 kg/m^2^). Patients with a history of OHSS, PCOS or poor response, were excluded. In the Requena et al. study [3], the oocyte donors included in the study aged 18–35 years, had regular menses and had a body weight > 60 kg with a BMI up to 29 kg/m^2^ with at least seven antral follicles at the beginning of the cycle. Donors having PCOS or multifollicular ovaries were excluded. The study of Kolibianakis et al. [16] included patients with previous poor ovarian response defined as retrieval of ≤4 COCs in a previous IVF cycle in which a starting dose of at least 450 IU per day was used, age <45 years, regular spontaneous menstrual cycle (24–35 days), body mass index (BMI) of 18–32 kg/m^2^ and basal FSH ≤20 IU/l. Differences among studies may represent sources of biases and, as it will be discussed later, influenced the result in terms of heterogeneity.

**Table 1:** Characteristics of studies included the meta-analysis of corifollitropin alfa versus recombinant FSH

Author and year	Study size (n1/n2)	RCT	Inclusion and exclusion criteria	Corifollitropin alfa protocol	Recombinant FSH protocol
Devroey et al, 2009 [12]	756/750	yes	Women aged 18–36 y with a body weight >60 kg up to and including 90 kg, a BMI of 18–32 kg/m^2^, a menstrual cycle length of 24-35 days, access to ejaculatory sperm, and an indication to COS before IVF or ICSI.	Patients started their treatment cycle on menstrual cycle day 2 or 3. Single s.c. injection 150 μg corifollitropin alfa, or matching placebo. From stimulation day 8 onwards treatment was continued with a daily s.c. of rFSH up to and including the day of hCG administration. The maximum rFSH dose to continue treatment after the first 7 days was for 7 d + ≤ 200 IU (from sd 8) rFSH + GnRH antagonist (ganirelix, 0.25 mg). 5000–10,000 IU urinary hCG. Progesterone ≥600 mg/d vaginally or at least 50 mg/d IM.	Placebo + 200 IU rFSH + 200 IU (from sd 8) rFSH + GnRH antagonist (ganirelix, 0.25 mg). 5000–10,000 IU urinary hCG. P ≥600 mg/d vaginally or at least 50 mg/d IM.
			Exclusion criteria: endocrine abnormality, abnormal blood biochemistry or hematology, abnormal cervical smear, chronic disease, relevant ovarian, tubal or uterine pathology that could interfere with COS embryo implantation or pregnancy, history of ovarian hyper-response (>30 follicles >11 mm) or OHSS, PCOS, or basal AFC >20 on ultrasound, or women with history of low ovarian response to FSH or hMG treatment, basal FSH or LH >12 IU/L in early follicular phase, >3 consecutive unsuccessful IVF cycles, history of ≥3 recurrent miscarriages, smoking >5 cigarettes per day.		
Ensure, 2010 [14]	268/128	yes	Women aged 18–36 y with body weight ≤60 kg, BMI 18–32 kg/m^2^, normal menstrual cycle length (24–35 days).	Single injection 100 μg corifollitropin alfa SC + placebo for 7 d + ≤ 200 IU (from sd 8) rFSH + GnRH antagonist (ganirelix, 0.25 mg). 5000–10,000 IU urinary hCG. Progesterone ≥600 mg/d vaginally or at least 50 mg/d IM.	Placebo + 200 IU rFSH (follitropin beta) + ≤200 IU (from sd 8) rFSH + GnRH antagonist (ganirelix, 0.25 mg). 5000–10,000 IU urinary hCG. Progesterone ≥600 mg/d vaginally or ≥50 mg/d IM.
			Exclusion criteria: The same as those reported in the Engage trial.		
Corifollitropin Alfa Dose-Finding Study Group, 2008 [9]	242/83	yes	Women aged 20–39 y with a normal menstrual cycle (24–35 days) and a BMI 17–31 kg/m^2^.	Single SC dose of 60, 120, or 180 μg corifollitropin alfa + 150 IU (from sd 8) rFSH (follitropin beta) + GnRH antagonist (ganirelix, 0.25 mg) up to the day of hCG administration (10,000 IU). Daily progesterone to support luteal phase.	150 IU rFSH + GnRH antagonist (ganirelix, 0.25 mg) up to the day of hCG administration (10,000 IU) 10,000 IU hCG. Daily progesterone to support luteal phase.
			Exclusion criteria: history of OHSS, PCOS, or any endocrine abnormality, previous poor response to FSH or hMG, more than 3 unsuccessful COS cycles, fewer than 2 ovaries, abnormal hormone levels during days 2–7 of menstrual cycle, use of hormonal preparations within 1 month before treatment or previous use of corifollitropin alfa.		
Devroey et al., 2004 [15]	75/24	yes	Women aged 18–39 y, BMI 18–29 kg/m^2^, with a regular menstrual cycle (24–35 days).	Single SC dose of corifollitropin alfa) of 120, 180, or 240 μg followed one week later by 150 IU rFSH + GnRH antagonist (ganirelix, 0.25 mg). 10,000 IU of hCG. Vaginal micronized P (600 mg/d) or IM P (≥50 mg/d).	150 IU rFSH + GnRH antagonist (ganirelix, 0.25 mg) starting on the day that the leading follicle had reached 14 mm. 10,000 IU urinary hCG. Vaginal micronized P (600 mg/d) or IM progesterone (≥50 mg/d).
			Exclusion criteria: Not indicated		
Requena et al., 2013 [3]	63/68	yes	Oocyte donors aged 18-35 y with a regular menstrual cycle, no hereditary or chromosomal diseases, normal karyotype, at least 7 antral follicles at the beginning of the cycle, body weight ≥60 kg and BMI ≤29 kg/m^2^.	Oral contraceptive pill for a maximum of 21 days preceded ovarian stimulation. Single injection 150 μg corifollitropin alfa. From stimulation day 8 onwards treatment was continued with a daily s.c. of rFSH 200 IU if needed. GnRH antagonist (ganirelix, 0.25 mg). A single dose of GnRH agonist (0.1 mg Decapeptyl) to trigger final oocyte maturation.	Oral contraceptive pill for a maximum of 21 days preceded ovarian stimulation. 200 IU rFSH + GnRH antagonist (ganirelix, 0.25 mg). A single dose of GnRH agonist (0.1 mg Decapeptyl) to trigger final oocyte maturation
			Exclusion criteria: Oocyte donors who had PCOS based on Rotterdam criteria or multifollicular ovaries.		
Kolibianakis et al., 2015 [16]	40/39	yes	Women poor responder aged <45 y. Characteristics of corifollitropin alfa versus daily FSH group: mean age (40.1 ± 3.3 vs. 40.1 ± 3.7 years, respectively; p = 0.96), mean BMI (26.1 ± 3.4 vs. 26.2 ± 3.5 Kg/m^2^, respectively; p = 0.95), mean basal FSH (12.3 ± 4.6 vs. 11.1 ± 3.2 IU/L, respectively; p = 0.46).	Single dose of 150 μg (0.5 mL) corifollitropin alfa + GnRH antagonist (from sd 5 onwards) until hCG + 250 μg of rehCG + daily dose of recFSH (450 IU/day) (from sd8 until the day of hCG, if necessary) + vaginal micronized progesterone (600 mg/day).	Previous IVF cycle with a starting dose of at least 450 IU per day + GnRH antagonist (from sd5 onwards) until hCG + 250 μg of rehCG + daily dose of recFSH (450 IU/day) (from sd8 until the day of hCG, if necessary) + vaginal micronized progesterone (600 mg/day).
Boostanfar et al, 2012 [17] (Pursue trial)	694/696	yes	Women aged 35–42 years.	During the first 7 days of ovarian stimulation, single injection of 150 μg CFA. When required, they continued the cycle with daily rFSH (maximally 300 IU) until 3 follicles reached ≥17 mm. Ganirelix acetate (0.25 mg) was started on stimulation day 5 and recombinant human chorionic gonadotropin was given to trigger oocyte maturation. Three days after oocyte pick-up, 2 embryos were transferred.	Daily 300 IU rFSH for 7 days. When required, they continued the cycle with daily rFSH (maximally 300 IU) until 3 follicles reached ≥17 mm. Ganirelix acetate (0.25 mg) was started on stimulation day 5 and recombinant human chorionic gonadotropin was given to trigger oocyte maturation. Three days after oocyte pick-up, 2 embryos were transferred.

Note: *CFA* corifollitropin alfa; *AFC* antral follicle count; *BMI* body mass index; *COS* controlled ovarian stimulation; *CTP* C-terminal peptide; *IM* intramuscular; *MII* metaphase II; *OHSS* ovarian hyperstimulation syndrome; *PCOS* polycystic ovary syndrome; *SC* subctaneous; *sd* stimulation day

Pooling the results of these seven RCTs, there was no significant difference in terms of live birth rate (4 RCTs; OR 1.12, 95 % CI 0.96–1.31; P = .15; substantial heterogeneity: I^2^ = 70.09 %), clinical pregnancy rate (4 RCTs; OR 1.01, 95 % CI 0.84–1.20; P = .95; moderate heterogeneity: I^2^ = 40.43 %), and ongoing pregnancy rate (6 RCTs; OR 1.04, 95 % IC 0.90–1.20; P = .62; substantial heterogeneity: I^2^ = 63.40 %) between the two groups. Four studies reported the early miscarriage rate per randomized woman. No significant difference was found in this respect between patients treated with corifollitropin alfa and daily rFSH (4 RCTs; OR 1.20, 95 % CI 0.75–1.92; P = .45; low heterogeneity: I^2^ = 1.53 %). A significantly higher number of embryos was obtained in women treated with corifollitropin alfa if compared to the rFSH group (6 RCTs; WMD 0.77, 95 % CI 0.41–1.13; *P* < .0001; substantial heterogeneity: I^2^ = 67.21 %, H^2^ = 3.05). No significant difference was observed in the mean duration of stimulation (4 RCTs; WMD 0.36, 95 % CI −0.36–1.09; P = .32; considerable heterogeneity: I^2^ = 85.42 %, H^2^ = 6.86). Regarding the number of oocytes, meta-analysis of the six RTCs demonstrated a significantly higher number of oocytes retrieved in the corifollitropin alfa group in comparison to the daily rFSH group (7 RCTs; WMD 1.37, 95 % CI 0.58–2.16; P = .0007; substantial heterogeneity: I^2^ = 76.65 %, H^2^ = 4.28). Data relative to the number of mature (MII) oocytes were reported by the six RTCs. Overall, there was a significantly higher number of mature oocytes (MII) in the corifollitropin alfa in comparison to rFSH (6 RCTs; WMD 1.54, 95 % CI 0.66–2.43; P = .0006; substantial heterogeneity: I^2^ = 67.21 %, H^2^ = 6.23). Cancellation of the cycle due to low response (3 RCTs; OR 1.08, 95 % CI 0.69–1.67; P = .74; no heterogeneity: I^2^ = 0 %) was comparable between the two groups. Cycle cancellation due to high response (3 RCTs; OR 3.19, 95 % CI 1.07–9.45; P = .03; moderate heterogeneity: I^2^ = 44.24 %) was significantly higher in the corifollitropin alfa group than in daily rFSH group. The incidence of OHSS was comparable between the two study groups (5 RCTs; OR 1.03, 95 % CI 0.74–1.45; P = .84; no heterogeneity: I^2^ = 0 %).

## Discussion

Controlled ovarian stimulation for IVF has relied, since its introduction, on daily administration of various formulations of gonadotropins. A single injection of the long lasting FSH corifollitropin alfa can replace the first standard seven daily injections of gonadotropins. This may result, in line with current expectations in IVF, in a simplification of the treatment regimen and reduced treatment burden [1–3]. Corifollitropin alfa is administered as a single injection during menstrual cycle days 2 or 3 (stimulation day 1). Daily injections of FSH are started on stimulation day 8 if needed. A gonadotropin releasing hormone antagonist is associated starting on stimulation day 5 to prevent premature LH surge. In the current study, available RCTs were included in a meta-analysis comparing corifollitropin alfa with conventional daily injections of FSH in IVF patients. A substantial amount of data derived from two large phase III randomized, double dummy, double blind trials designed by the manufacturer, and including patient less than 36 years that were considered not at risk of developing OHSS (antral follicle count ≤ 20). There were 756 women weighting > 60 kg that received corifollitropin alfa at 150 μg [12, 13], and 268 with a body weight ≤ 60 kg that received 100 μg [14]. Results were compared with reference groups receiving daily injections of rFSH at 150 IU [15] or 200 IU [12, 13]. The Corifollitropin Alfa Dose Finding Study Group [8], a phase II study including 325 women, was undertaken to investigate the dose–response relationship with respect to the number of oocytes retrieved after administration of corifollitropin alfa at 60, 120 or 180 μg. Devroey et al. [15], in their phase II trial, randomized IVF patients to receive 120 μg (n = 25), 180 μg (n = 24), or 240 μg (n = 25) corifollitropin alfa or 150 IU of daily rFSH. The follow-up trial is a phase 3 randomized, double-blind, double-dummy, active-controlled, noninferiority trial, including women aged 35–42 years [17]. 1390 women were randomized to a single injection of 150 μg corifollitropin alfa (n = 694) or daily 300 IU rFSH during the first seen days of ovarian stimulation. A GnRH antagonist was started on stimulation day 5. When required, ovarian stimulation was continued with daily injections of rFSH at the maximal dose of 300 IU. As result, corifollitropin alfa was found to be non-inferior to rFSH in terms of effectiveness. Requena and co-workers [3] evaluated the degree of satisfaction in oocyte donors undergoing treatment with corifollitropin alfa (n = 48) compared with women receiving rFSH (n = 40). The study involved patients weighting more than 60 kg and thus receiving corifollitropin alfa at 150 μg. Assignment to each treatment group was made according to a quasi-experimental design which included consecutive opportunity sampling. Patients were pre-treated with an oral contraceptive pill before starting COS. Potential high responders were excluded. Moreover, to further reduce the risk of OHSS, GnRH agonist (instead of hCG) was used to trigger final oocyte maturation. The study carried out by Kolibianakis by et al. [16], included seventy-nine women and aimed to evaluate the potential benefit of corifollitropin alfa treatment compared to daily rFSH in poor responder patients. In this prospective, randomized, open label, non-inferiority clinical trial, women were considered poor responders if they had a previous poor response to ovarian stimulation. Other inclusion criteria were: age less than 45; regular menses; body mass index (BMI) of 18–32 kg/m2; and basal FSH ≤20 IU/l. On day 2 of menses women received either a single dose of corifollitropin alfa at 150 μg. When needed, 450 IU of rFSH were given from day 8 of stimulation up the hCG day. Results were compared with control groups receiving daily injections of rFSH at 450 IU. As a major finding, the number of cumulus oocyte complex that were recovered was not statistically different between the corifollitropin alfa and rFSH groups.

Pooling data from these studies showed no significant differences in the majority of clinical parameters considered between corifollitropin alfa and rFSH. This meta-analysis revealed comparable outcomes in terms of live birth rate, ongoing pregnancy rate and clinical pregnancy rate. Pregnancies obtained in women undergoing COS with corifollitropin alfa had the same risk of spontaneous abortion as pregnancies from rFSH. When the ovarian response to COS was considered, corifollitropin alfa yielded a higher number of oocytes than rFSH. In line with this finding, the number of embryos obtained per woman randomized in the corifollitropin alfa group was also higher. These outcomes, along with the evidence that a higher number of women treated with this drug experienced cycle cancellation due to overstimulation, underscore the need for a careful selection of the patients that are eligible for corifollitropin alfa in order to prevent OHSS. However, the major ovarian response associated to COS with corifollitropin alfa did not result in a statistically significant increased risk of OHSS. When discussing the risk of OHSS, it appears important to consider the criteria for cycle cancellation used. These are available for the Ensure and Engage RTCs. In both studies, the investigator was allowed to withhold rFSH administration for a maximum of 3 days. When the investigator considered the ovarian response too high, he was allowed to cancel the cycle at any time. In the case of a risk for OHSS (more than 30 follicles > 11 m on ultrasound scan) hCG was to be withheld and the treatment cycle would be cancelled. The Kolibianakis et al. [16] study compared corifollitropin alfa with rFSH in poor responder patients. Thus, there was no risk of OHSS. There were no significant differences with respect of length of stimulation associated to corifollitropin alfa in comparison to rFSH. Corifollitropin alfa has been designed to relieve the treatment burden experienced by IVF women. Results offered by the pooled analysis of this meta-analysis are compatible with the notion that proper exposure to corifollitropin alfa results in IVF outcome comparable to that observed with rFSH. This concept is in line with conclusions provided by two previously published meta-analyses [10, 11]. As a novel aspect, this meta-analysis included RCTs on two specific subpopulations of IVF patients, i.e. egg donors [3] and poor responders [16]. Conclusions of our meta-analysis could be slightly different when separately considering these subpopulations. Unfortunately, the number of this type of studies is not sufficient to perform a population-specific meta-analysis. This circumstance is reflected by moderately higher levels of heterogeneity, as seen in the previous section. To satisfyingly detect it, we need to consider that H^2^ indexes in the above experiments are always significantly bigger than one, and this is sign of strong diversity between groups. The resulting analysis may be thus more reflective of the everyday clinical practice. Also consider that at the time of writing only a limited number of RCTs was available. This represented a major limitation of this meta-analysis. For instance we were unable to analyze publication bias using funnel plots, since it is known that at least ten RCTs are required to get statistically significant results [18]. A concept that must be born in mind when using corifollitropin alfa is that its unique pharmacokinetic profile can result in the recruitment of an increased cohort of developing follicles, and this contraindicates its use in high responder patients that are at risk of OHSS. Adequate definition of the ovarian reserve and thus exclusion of patients with high ovarian reserve appears to remain a crucial step before considering long lasting FSH for COS.

## Conclusions

Corifollitropin alfa was designed to simplify the treatment regimen in patients undergoing ovarian stimulation for IVF. This systematic review and meta-analysis suggests that corifollitropin alfa is effective as rFSH in terms of live birth rate, ongoing pregnancy rate and clinical pregnancy rate. The increased number of eggs retrieved under corifollitropin alfa regimen reflects the elevated effectiveness of this novel FSH formulation, but warns at the same time against the possible increased risk of OHSS in high responder women.

## Abbreviations

CI: Confidence interval
COS: Controlled ovarian stimulation
ICSI: Intracytoplasmic sperm injection
IVF: In vitro fertilization
OHSS: Ovarian hyperstimulation syndrome
OR: Odds ratios
rFSH: Recombinant follicle stimulating hormone
RTCs: Randomized controlled trials
WMD: Weighted mean differences
